# The Association Between Structural Disadvantage and Adverse Birth Outcomes: Analyzing Preterm Birth and Low Birth Weight Using the Structural Racism Effect Index

**DOI:** 10.1007/s40615-025-02454-1

**Published:** 2025-04-29

**Authors:** Emma A. Blackson, Briana Williams, Jé Judson, Caryn Bell, Maeve Wallace

**Affiliations:** 1 Department of Social, Behavioral, and Population Sciences, Celia Scott Weatherhead School of Public Health & Tropical Medicine, Tulane University, New Orleans, Louisiana, USA; 2 Division of Epidemiology and Community Health, University of Minnesota, Minneapolis, Minnesota, USA; 3 Department of International Health and Sustainable Development, Celia Scott Weatherhead School of Public Health & Tropical Medicine, Tulane University, New Orleans, Louisiana, USA; 4 Department of Health Promotion Sciences, Mel and Enid Zuckerman College of Public Health, University of Arizona, Tucson, Arizona, USA

**Keywords:** Structural disadvantage, Neonatal health, Health disparities, Low birth weight, Preterm birth

## Abstract

Patterns of risk for adverse birth outcomes, including low birth weight and preterm birth, are influenced by structural disadvantage. The Structural Racism Effect Index (SREI) measures these disadvantages across nine different domains, including education, income, housing, and employment. This study examines the association between structural disadvantage, as measured by the SREI, and adverse birth outcomes in the USA. Using the National Center for Health Statistics’ 2020 natality file, modified Poisson regression models with robust variance adjusted for maternal age, education, prenatal care initiation, previous preterm birth, and payment source estimated the relative risks of low birth weight and preterm birth associated with exposure to level of SREI at the maternal county of residence. Among 2,376,030 birth records, higher SREI scores were significantly associated with increased risks of adverse outcomes. Medium and high SREI levels were linked to 7.4% (95% CI 6.3–8.5%) and 18.8% (95% CI 16.9–20.8%) higher risks for preterm birth, respectively, and 6.1% (95% CI 4.9–7.2%) and 17.0% (95% CI 15.8–18.2%) higher risks for low birth weight, respectively, compared to low SREI scores. Stratified analyses revealed that the impact of SREI on adverse birth outcomes varied significantly by maternal race, highlighting differential effects across racial groups. Cuzick’s test for linear trend confirmed the dose–response relationship between the extent of structural disadvantage and the likelihood of adverse birth outcomes.

## Background

Structural racism refers to the interwoven policies, institutional practices, and cultural norms that systematically reinforce racial inequities by shaping access to resources, opportunities, and health across generations [1, 2]. The Structural Racism Effect Index (SREI), developed by Dyer et al., is a tool for quantifying structural racism on health, aiming to improve upon existing measures by capturing the downstream consequences of structural racism across diverse communities within the USA [3]. However, the original study establishing the SREI did not assess its association with adverse birth outcomes, such as low birth weight and preterm birth. Adverse birth outcomes are influenced by multiple interrelated structural factors, including economic, educational, environmental, and social domains embedded within communities [4–6]. Previous literature highlights significant racial disparities in birth outcomes, with non-Hispanic Black individuals consistently experiencing higher rates of preterm birth and low birth weight compared to their non-Hispanic White counterparts [4, 5, 7, 8]. Initial validation analyses of the SREI revealed stark racialized patterns of structural disadvantage, with people of color comprising only 2.8% of census tracts in the least disadvantaged decile (decile 1), compared to 92.8% in the most disadvantaged decile (decile 10) [3]. While structural racism is recognized as a significant determinant of these inequities, empirical measures capturing the full breadth of structural disadvantage remain limited.

The SREI offers a comprehensive, race-neutral design that ensures the SREI measures broader structural deficits affecting various populations rather than focusing solely on racial inequities across multiple domains [3], specifically the built environment, criminal justice, education, employment, housing, income and poverty, social cohesion, transportation, and wealth [3]. By aggregating data at the community level, the SREI provides a robust measure of structural disadvantage that does not explicitly incorporate racial composition, enabling an examination of how place-based disadvantage affects health outcomes across racial and ethnic groups.

This current study aims to explore the association between structural disadvantage, measured by the SREI, and low birth weight and preterm birth among newborns in the USA. By spatially linking the SREI with county-level birth data from 2020, this research seeks to provide empirical evidence on how structural disadvantages at the county level influence neonatal health and contribute to persistent racial inequities. Through this study, we aim to contribute to the ongoing discourse on health equity by illustrating how structural disadvantages, regardless of their origins in racial discrimination, impact health outcomes across communities. This research will inform targeted public health interventions to mitigate the adverse effects of structural disadvantage.

In this article, we use the term *birthing people* to refer to individuals who give birth, acknowledging that not all individuals who experience pregnancy identify as mothers.

## Methods

### Data Sources

This study utilized two datasets. The SREI dataset includes census tract-level measures developed by Dyer et al. [3] and obtained from their website [9] dedicated to the SREI, of which the measure itself was developed across multiple domains as described above, compiled from sources including the US Census Bureau and other federal databases. Both total and domain-specific SREI scores were aggregated to the county level by taking the tract population mean across neighborhoods within each county. Data on all live births occurring in the USA in 2020 (*n* = 3,605,201) came from the National Center for Health Statistics restricted use natality file, which includes birth records with a geographic identifier (Federal Information Processing System [FIPS] code) for maternal county of residence. These data were linked to the county-aggregated SREI scores by county FIPS code for maternal county of residence, where available. We used 2020 birth data to align with close temporality to the SREI derived from 2015 to 2019 5-year census estimates. Any cases with missing values were deleted.

### Outcome

Outcome data for our variables of interest came from the natality file. Preterm birth was defined as birth occurring at < 37 weeks of gestation, while low birth weight was defined as birth weight < 2500 g. These were analyzed as separate, dichotomous variables.

### Exposure

County-level total SREI score was our primary exposure of interest. For ease of interpretation and to avoid assumptions of linear trend, we grouped counties into tertiles of SREI based on the sample distribution with low corresponding to counties with the smallest SREI values (i.e., the least amount of structural disadvantage), followed by medium and high (counties with the largest SREI values indicating the greatest amount of structural disadvantage).

### Covariates

In adjusted models, we controlled for characteristics identified a priori as related to risk for adverse birth outcomes and available on the birth record. These included maternal age (< 20, 20–24, 25–29, 30–34, ≥ 35), educational attainment (Basic Education, Some College, Bachelor’s degree, and Advanced Degree), payment source for delivery as a proxy for socioeconomic position (Medicaid, private insurance, self-pay, other), timing of prenatal care initiation (1 st–3rd month, 4 th–6 th month, 7 th month to birth, no prenatal care), and previous pre-term birth (yes, no).

### Statistical Analysis

We used descriptive statistics to characterize the births under study. We fit Log-Poisson regression models with robust standard errors to account for the clustering of births within counties. These models allow for the estimation of adjusted relative risk for adverse birth outcomes associated with living in medium and high levels of structural disadvantage and racism compared to the lowest levels. We also did the same to the domains that comprise the SREI to see which ones were more associated with our outcomes. To further demonstrate the contribution of the SREI to racial inequity in birth outcomes, we applied the approach proposed by Ward et al. [10]. Specifically, the approach involves (1) estimating racial differences in the prevalence of exposure to SREI, (2) estimating racial differences in adverse birth outcomes, and (3) testing for racial heterogeneity in the association between SREI and adverse birth outcomes [10]. Finally, we conducted a test for linear trend to confirm the dose–response relationship between SREI levels and adverse birth outcomes. We used Cuzick’s non-parametric test for trend to assess the dose–response relationship between SREI levels and adverse birth outcomes. Cuzick’s test was chosen over alternative trend tests, such as the chi-square for trend, due to its robustness to non-normality and its suitability for ordered categorical data. This non-parametric approach allowed us to confirm a consistent trend across SREI tertiles without assuming linearity in the effect, thereby supporting the hypothesis that greater structural disadvantage is associated with increased risk of adverse birth outcomes. Stata 18 was used to conduct these analyses.

### Ethics and Data Privacy

This study of existing, de-identified data was deemed not human subjects research as defined by the Federal Common Rule by the Tulane University Institutional Review Board.

## Results

### Descriptive Statistics of Study Participants and Prevalence by Race

Among the 2,376,030 births included in this study, 9.41% were preterm, and 7.72% were low birth weight (Table 1). Non-Hispanic White individuals constituted the majority of birthing people (51.3%), followed by Hispanic (22.9%), non-Hispanic Black (17.2%), and non-Hispanic Other (8.6%). Most birthing people (76.9%) initiated prenatal care within the first 3 months of pregnancy, with 37.5% having completed high school or a GED and 27.1% some college. Medicaid (41.9%) and private insurance (51.1%) were the most common payment sources for delivery. Non-Hispanic Black individuals and Medicaid-covered births were disproportionately overrepresented in counties with high SREI scores.

**Table 1:** Descriptive statistics by SREI categories

Variable	Category	Low SREI	Medium SREI	High SREI	Total
	Total	770,948	823,936	781,146	2,376,030
Preterm birth	No	706,532 (91.6%)	746,611 (90.7%)	699,262 (89.5%)	2,152,405 (90.6%)
	Yes	64,416 (8.4%)	77,325 (9.3%)	81,884 (10.5%)	223,625 (9.4%)
Low birth weight	No	718,424 (93.2%)	761,255 (92.4%)	712,951 (91.3%)	2,192,630 (92.3%)
	Yes	52,524 (6.8%)	62,681 (7.6%)	68,195 (8.7%)	183,400 (7.7%)
Maternal race	NH White	437,075 (56.7%)	396,289 (48.1%)	385,460 (49.3%)	1,218,824 (51.3%)
	NH Black	89,708 (11.6%)	138,829 (16.8%)	179,303 (22.9%)	407,840 (17.2%)
	Hispanic	148,560 (19.3%)	228,432 (27.7%)	166,716 (21.3%)	543,708 (22.9%)
	NH Other	95,605 (12.4%)	60,386 (7.3%)	49,667 (6.4%)	205,658 (8.6%)
Maternal age	< 20	18,772 (2.4%)	36,403 (4.4%)	49,120 (6.3%)	104,295 (4.4%)
	20–24	97,176 (12.6%)	147,522 (17.9%)	186,956 (23.9%)	431,654 (18.2%)
	25–29	195,162 (25.3%)	236,111 (28.7%)	240,475 (30.8%)	671,748 (28.3%)
	30–34	269,527 (35.0%)	248,761 (30.2%)	195,796 (25.1%)	714,084 (30.0%)
	35 +	190,311 (24.7%)	155,139 (18.8%)	108,799 (13.9%)	454,249 (19.1%)
Payment for birth	Medicaid	241,481 (31.3%)	347,403 (42.2%)	406,904 (52.1%)	995,788 (41.9%)
	Private insurance	483,858 (62.8%)	417,541 (50.7%)	313,499 (40.1%)	1,214,898 (51.1%)
	Self pay	21,413 (2.8%)	40,218 (4.9%)	29,019 (3.7%)	90,650 (3.8%)
	Other	24,196 (3.1%)	18,774 (2.3%)	31,724 (4.1%)	74,694 (3.1%)
Mother’s education	Basic education	213,960 (27.8%)	318,889 (38.7%)	357,509 (45.8%)	890,358 (37.5%)
	Some college	184,469 (23.9%)	223,065 (27.1%)	235,208 (30.1%)	642,742 (27.1%)
	Bachelor’s degree	216,956 (28.1%)	179,208 (21.7%)	124,049 (15.9%)	520,213 (21.9%)
	Advanced degree	155,563 (20.2%)	102,774 (12.5%)	64,380 (8.2%)	322,717 (13.6%)
Month prenatal care began	1 st to 3rd month	627,598 (81.4%)	621,187 (75.4%)	578,474 (74.1%)	1,827,259 (76.9%)
	4 th to 6 th month	109,506 (14.2%)	143,789 (17.5%)	147,262 (18.9%)	400,557 (16.9%)
	7 th to final month	27,208 (3.5%)	39,761 (4.8%)	38,442 (4.9%)	105,411 (4.4%)
	No prenatal care	6636 (0.9%)	19,199 (2.3%)	16,968 (2.2%)	42,803 (1.8%)
Previous preterm birth	No	745,132 (96.6%)	793,887 (96.4%)	746,358 (95.5%)	2,285,377 (96.2%)
	Yes	25,816 (3.4%)	30,049 (3.6%)	34,788 (4.5%)	90,653 (3.8%)

^*^Note that this table contains column percentages

Exposure to the SREI varied significantly by race/ethnicity (*χ*^2^ = 68,000; *p* < 0.001). Non-Hispanic Black individuals exhibited the highest prevalence of high SREI exposure (43.96%), followed by Hispanic (30.66%), non-Hispanic White (31.63%), and “other” racial/ethnic groups (24.15%). Medium SREI levels were most prevalent among Hispanic individuals (42.01%), while low SREI exposure was more common among the “other” category (46.49%).

Adverse birth outcomes also differed significantly by race/ethnicity. Non-Hispanic Black individuals exhibited the highest prevalence of preterm birth (12.83%) and low birth weight (12.78%) compared to non-Hispanic White (8.44% and 6.29%), Hispanic (9.30% and 6.93%), and “other” racial/ethnic groups (8.67% and 8.25%) (*χ*^2^ = 7100 and *χ*^2^ = 19,000; *p* < 0.001 for both). Crude prevalence estimates showed that counties with high SREI scores had higher rates of preterm birth (10.5%) and low birth weight (8.7%) compared to counties with low SREI scores (8.4% and 6.8%, respectively).

### Poisson Regression Crude and Multivariate Analyses

Tables 2, 3, and 4 present the results of the Poisson regression analyses examining associations between SREI tertiles and adverse birth outcomes. Counties with high SREI scores were associated with a significantly increased risk of preterm birth (18.8%; 95% CI 16.9–20.8%) and low birth weight (17.0%; 95% CI 15.8–18.2%) compared to low SREI areas among adjusted models (see Table 3 and 4). Medium SREI levels were associated with a smaller but still significant increased risk of preterm birth (7.4%; 95% CI 6.3–8.5%) and low birth weight (6.1%; 95% CI 4.9–7.2%).

**Table 2:** Poisson regression analysis of SREI domains and preterm birth and low birthweight

SREI domain	SREI category	Preterm birth RR (95% CI)	Low birthweight RR (95% CI)
Built environment	Medium	1.019 (1.005, 1.032)**	1.029 (1.015, 1.044)**
	High	1.078 (1.059, 1.091)**	1.088 (1.067, 1.103)**
Criminal justice	Medium	1.012 (1.001, 1.023)**	1.038 (1.027, 1.050)**
	High	1.058 (1.045, 1.070)**	1.101 (1.083, 1.110)
Education	Medium	1.029 (1.016, 1.043)**	0.992 (0.977, 1.006)
	High	1.044 (1.028, 1.058)**	0.946 (0.927, 0.962)**
Employment	Medium	1.050 (1.037, 1.060)**	1.010 (0.998, 1.023)
	High	1.075 (1.058, 1.088)**	1.053 (1.034, 1.069)**
Housing	Medium	0.981 (0.969, 0.993)**	1.015 (1.002, 1.029)**
	High	1.000 (0.986, 1.014)	1.072 (1.054, 1.085)**
Income and poverty	Medium	0.943 (0.929, 0.957)**	0.950 (0.932, 0.964)**
	High	0.953 (0.935, 0.970)**	0.995 (0.976, 1.015)
Social cohesion	Medium	1.045 (1.031, 1.056)**	1.063 (1.047, 1.075)**
	High	1.114 (1.093, 1.125)**	1.152 (1.125, 1.158)**
Transportation	Medium	1.046 (1.031, 1.059)	1.036 (1.019, 1.051)**
	High	1.030 (1.011, 1.048)	1.029 (1.008, 1.049)**
Wealth	Medium	1.003 (0.989, 1.017)	0.988 (0.972, 1.003)
	High	1.026 (1.006, 1.045)	1.004 (0.982, 1.026)

** indicates statistical significance at the 0.05 level. The RRs are exponentiated coefficients from the Poisson regression models, and the 95% CIs are derived from the robust standard errors

**Table 3:** Crude and adjusted relative risk and 95% CI for preterm birth crude and adjusted relative risk and 95% CI for preterm birth associated with county-level SREI

Group	SREI category	Crude RR	95% CI	Adjusted RR	95% CI adjusted
Total population	Low	Reference	-	Reference	-
	Medium	1.12**	1.11, 1.13	1.09**	1.07, 1.10
	High	1.25**	1.24, 1.27	1.18**	1.16, 1.19
NH White	Low	Reference	-	Reference	-
	Medium	1.10**	1.09, 1.11	1.08**	1.06, 1.09
	High	1.23**	1.22, 1.24	1.16**	1.14, 1.17
NH Black	Low	Reference	-	Reference	-
	Medium	1.14**	1.12, 1.16	1.14**	1.11, 1.16
	High	1.24**	1.21, 1.26	1.22**	1.19, 1.24
Hispanic	Low	Reference	-	Reference	-
	Medium	1.04**	1.02, 1.06	1.04**	1.02, 1.06
	High	1.10**	1.08, 1.12	1.11**	1.08, 1.13
Other	Low	Reference	-	Reference	-
	Medium	1.06**	1.03, 1.09	1.04**	1.01, 1.07
	High	1.15**	1.12, 1.18	1.08**	1.05, 1.11

^*^Adjusted models included maternal age, educational attainment, payment source, timing of prenatal care, and previous preterm birth

** indicates statistical significance at the 0.05 level. The RRs are exponentiated coefficients from the Poisson regression models, and the 95% CIs are derived from the robust standard errors

**Table 4:** Crude and adjusted relative risk and 95% CI for low birth weight associated with county-level SREI

Group	SREI category	Crude RR	95% CI	Adjusted RR	95% CI adjusted
Total population	Low	Reference	-	Reference	-
	Medium	1.11**	1.10, 1.12	1.06**	1.05, 1.07
	High	1.28**	1.27, 1.29	1.17**	1.16, 1.18
NH White	Low	Reference	-	Reference	-
	Medium	1.10**	1.08, 1.12	1.05**	1.03, 1.06
	High	1.23**	1.21, 1.25	1.12**	1.10, 1.14
NH Black	Low	Reference	-	Reference	-
	Medium	1.15**	1.13, 1.17	1.12**	1.10, 1.14
	High	1.26**	1.23, 1.28	1.20**	1.17, 1.23
Hispanic	Low	Reference	-	Reference	-
	Medium	1.00	0.98, 1.02	1.01	0.98, 1.03
	High	1.07**	1.05, 1.10	1.06**	1.03, 1.08
Other	Low	Reference	-	Reference	-
	Medium	1.02	0.99, 1.05	1.01	0.97, 1.04
	High	1.06**	1.03, 1.10	1.05**	1.02, 1.08

^*^Adjusted models included maternal age, educational attainment, payment source, timing of prenatal care, and previous preterm birth

** indicates statistical significance at the 0.05 level. The RRs are exponentiated coefficients from the Poisson regression models, and the 95% CIs are derived from the robust standard errors

The social cohesion domain of the SREI showed particularly strong associations with adverse outcomes, with high levels linked to a 10.8% higher risk of preterm birth (95% CI 9.3–12.3%) and a 14.1% higher risk of low birth weight (95% CI 12.5–15.8%) (Table 2). Conversely, high levels of income and poverty disadvantage were inversely associated with preterm birth, suggesting potential protective effects in certain low-income communities.

Predicted margins analysis revealed that non-Hispanic Black individuals consistently exhibited the highest relative risks for preterm birth and low birth weight across all SREI tertiles. For example, in high SREI counties, the predicted preterm birth margin was 12.66% for non-Hispanic Black individuals compared to 9.51% for non-Hispanic Whites. Margin analysis for low birth weight showed parallel trends, with non-Hispanic Black individuals exhibiting the highest relative risks within each SREI tertile (see Tables 5 and 6).

**Table 5:** Multivariate models with interaction term to assess differential effects

Maternal race	SREI category	RR for preterm birth (95% CI)	RR for low birth weight (95% CI)
Non-Hispanic White	Low	Reference	Reference
	Medium	1.08 (1.07, 1.10)**	1.07 (1.05, 1.09)**
	High	1.18 (1.16, 1.20)**	1.16 (1.13, 1.18)**
Non-Hispanic Black	Low	1.32 (1.30, 1.34)**	1.71 (1.66, 1.75)**
	Medium	1.36 (1.30, 1.66)**	1.72 (1.51, 1.94)**
	High	1.18 (1.15, 1.23)**	1.57 (1.50, 1.67)**
Hispanic	Low	1.16 (1.13, 1.20)**	1.06 (1.03, 1.10)**
	Medium	0.95 (0.93, 0.98)**	0.92 (0.90, 0.95)**
	High	1.07 (1.06, 1.10)**	1.02 (1.01, 1.06)**
Other	Low	1.16 (1.14, 1.18)**	1.16 (1.13, 1.20)**
	Medium	0.91 (0.87, 0.98)**	0.93 (0.90, 0.96)**
	High	0.92 (0.88, 0.95)**	0.82 (0.79, 0.85)**

^*^Adjusted models included maternal age, educational attainment, payment source, timing of prenatal care, and previous preterm birth

** indicates statistical significance at the 0.05 level. The RRs are exponentiated coefficients from the Poisson regression models, and the 95% CIs are derived from the robust standard errors

**Table 6:** Relative risks of preterm birth and low birth weight by race and SREI tertile

Maternal race	SREI category	Preterm birth margin	95% CI (preterm birth)	*p*-value (preterm birth)	Low birth weight margin	95% CI (low birth weight)
Non-Hispanic White	Low	0.0804	(0.0795, 0.0812)	< 0.001	0.0613	(0.0605, 0.0620)
	Medium	0.0871	(0.0862, 0.0880)	< 0.001	0.0656	(0.0648, 0.0664)
	High	0.0951	(0.0941, 0.0960)	< 0.001	0.0711	(0.0703, 0.0719)
Non-Hispanic Black	Low	0.1060	(0.1040, 0.1079)	< 0.001	0.1046	(0.1026, 0.1065)
	Medium	0.1192	(0.1175, 0.1208)	< 0.001	0.1169	(0.1153, 0.1186)
	High	0.1266	(0.1251, 0.1281)	< 0.001	0.1252	(0.1237, 0.1267)
Hispanic	Low	0.0865	(0.0851, 0.0879)	< 0.001	0.0653	(0.0641, 0.0665)
	Medium	0.0895	(0.0884, 0.0907)	< 0.001	0.0645	(0.0635, 0.0655)
	High	0.0947	(0.0933, 0.0961)	< 0.001	0.0685	(0.0673, 0.0696)
Other	Low	0.0861	(0.0843, 0.0879)	< 0.001	0.0861	(0.0843, 0.0880)
	Medium	0.0897	(0.0874, 0.0920)	< 0.001	0.0855	(0.0832, 0.0878)
	High	0.0940	(0.0915, 0.0966)	< 0.001	0.0826	(0.0802, 0.0850)

**Note:** The margins represent the predicted probability of each outcome (preterm birth and low birth weight) across racial and SREI groups, with 95% confidence intervals calculated using robust standard errors

### Dose–Response Relationship

Cuzick’s non-parametric trend test confirmed a significant dose–response relationship for preterm birth across increasing SREI tertiles (*z* = 45.381; *p* < 0.001), indicating that higher SREI levels were consistently associated with higher prevalence of preterm birth. A similar trend was observed for low birth weight (*z* = 44.764; *p* < 0.001) (see Table 7).

**Table 7:** Dose–response relationship

Outcome	SREI category	Mean response score	Number of observations	Test statistic	Std. error	*z*-Score	*p*-value
Preterm birth	Low	0.0836	770,948				
	Medium	0.0938	823,936				
	High	0.1048	781,146	8254.097	181.8835	45.381	< 0.001
Low birth weight	Low	0.0681	770,948				
	Medium	0.0761	823,936				
	High	0.0873	781,146	7441.921	166.2468	44.764	< 0.001

The *p*-values (< 0.001) indicate statistical significance at the 0.05 level for the trend test, suggesting a significant dose–response relationship between SREI categories and the outcomes of preterm birth and low birth weight

## Discussion

In this cross-sectional analysis, the adjusted multivariate Poisson regression models confirm the robustness of the association between the SREI and adverse birth outcomes, specifically preterm birth and low birth weight. Higher maternal age and previous preterm birth emerged as strongly associated with adverse outcomes, while advanced education and private insurance were protective against preterm birth and low birth weight. Interestingly, income and poverty disadvantage showed an inverse association with preterm birth in some analyses. This pattern may reflect complex socioeconomic dynamics within certain low-income areas, including possible support structures or community services that mitigate some of the risks of adverse birth outcomes.

The observed dose–response relationship across SREI tertiles validated the index as a meaningful tool for identifying structural disadvantage and health risks. As SREI scores increased, so did the likelihood of preterm birth and low birth weight (Table 7). To confirm this relationship, we conducted a test for linear trend, which showed a significant trend, indicating that as the level of structural disadvantage increases, so does the likelihood of adverse birth outcomes. Further, by applying the approach proposed by Ward et al. [10], we demonstrated that exposure to high levels of structural disadvantage, as measured by the SREI, was disproportionately experienced by Black birthing people, consistent with our bivariate and multivariate results. The association between SREI and adverse birth outcomes varied by race, reinforcing the role of structural disadvantage in shaping persistent racial disparities in maternal and infant health. These results further emphasize the need for structural interventions that address place-based inequities rather than solely focusing on individual-level risk factors to mitigate disparities in birth outcomes.

Notably, differential effects across racial and ethnic groups indicate that structural disadvantage intersects uniquely with community-specific social contexts. However, among non-Hispanic Black birthing people, we did not observe a clear dose–response relationship for preterm birth across SREI tertiles. This suggests that their elevated risk remains consistently high, likely due to the cumulative effects of systemic racism that extend beyond neighborhood-level disadvantage. Chronic exposure to racism-related stressors, such as discrimination and weathering, may create persistent risks not fully captured by the SREI. These findings highlight the need for tools that directly account for race-specific structural and psychosocial burdens.

Our findings underscore structural conditions, not individual racial characteristics, are key drivers of poor health outcomes. That the SREI predicts poor outcomes in predominantly White regions such as Appalachia [3] highlight how structural disinvestment, economic exclusion, and policy-driven neglect are critical determinants of health, regardless of racial composition. However, this should not be conflated with the idea that structural racism is simply a function of poverty. In the USA, racism has been a central organizing force in the distribution of resources, opportunities, and systemic harms, with Black, Indigenous, and other marginalized groups disproportionately affected [2, 11, 12].

While the SREI is a powerful tool for assessing place-based inequities, it captures structural disadvantage more broadly rather than isolating race-specific mechanisms of oppression. The racialized patterns in our results suggest that structural racism intersects with, but is not synonymous with, general structural disadvantage. To better understand these dynamics, we stratified our models by race, allowing us to examine how structural conditions operate differently across racial groups. Future refinements to the SREI could help distinguish structural racism more directly, for example, by incorporating race-sensitive indicators such as disparities in law enforcement or access to culturally competent healthcare. These additions may help clarify how racism, beyond general disadvantage, uniquely shapes health risks, especially for non-Hispanic Black populations who face elevated rates of adverse birth outcomes even when controlling for socioeconomic factors. Ultimately, these findings affirm the importance of structural determinants in shaping health outcomes from the earliest stages of life and highlight the need for tools that disentangle the overlapping but distinct impacts of race and class-based inequity.

Other studies demonstrating the pervasive impact of structural disadvantage and racism on maternal and infant health confirm the need for targeted public health interventions and policies to mitigate these effects [5, 13–16]. Our findings highlight similar magnitudes of association between structural disadvantage and adverse birth outcomes compared to previous studies. Additionally, geographic variations in SREI scores and health outcomes emphasize the importance of local context in these associations.

### Public Health, Clinical, Political, and Social Justice Implications

The findings from this study have profound implications for public health and clinical, political, and social justice. Public health initiatives should be tailored to address the specific needs of communities with varying SREI scores, recognizing that structural disadvantage’s impact is not uniform across racial/ethnic groups. For example, targeted interventions for non-Hispanic Black birthing people in high SREI areas could focus on culturally competent community health programs designed to mitigate elevated risks of adverse birth outcomes. These racially and contextually tailored approaches could increase the effectiveness of public health strategies by aligning resources with the specific risk patterns and resilience factors within each group.

These findings highlight the need for healthcare providers to consider structural and social determinants in clinical care, integrating contextual factors into assessments and treatment plans [17, 18]. Addressing health disparities also requires policy efforts that tackle inequities in housing, education, and economic opportunity. Local governments and lawmakers can use these insights to guide investments and zoning policies that improve conditions in disadvantaged areas [19–21]. Partnering with community organizations, local health departments can ensure interventions are both culturally relevant and accessible.

Finally, this study highlights the urgency of addressing the root causes of health disparities by advocating for equitable resource distribution and dismantling systemic barriers rooted in structural racism and oppression. Government entities, corporations, and media institutions must use their influence and investments to disrupt systems perpetuating inequities. By embedding social justice into public health strategies, we can move toward eliminating disparities, not only in health, but across the broader societal structures that shape well-being. When the most marginalized communities are supported in living full, healthy lives, all of society benefits.

### Limitations and Future Directions

Several limitations warrant consideration, including the potential underestimation of localized impacts of structural disadvantage due to geographical variations and the reliance on cross-sectional data from 2020, which limits causal inferences. The COVID-19 pandemic, occurring in the same year as our data, may have influenced socioeconomic indicators, access to care, and other aspects of the SREI. COVID-19 differentially impacted racial and ethnic groups [22, 23], which could contribute to our findings and should be acknowledged as a potential limitation. Additionally, while we adjusted for known confounders, residual confounding is possible, particularly regarding unmeasured environmental exposures or individual health behaviors that the SREI and birth records do not capture. This is particularly relevant given the observed racial/ethnic variations, which could partially reflect unmeasured social or environmental differences not fully accounted for in this study. Finally, we have no data on the duration of residence for the maternal county of residence, and in such cases where a person moved to a different county at some point during their pregnancy, there is the possibility of exposure misclassification.

Future studies using the SREI would benefit from explicitly incorporating Critical Race Theory (CRT) [24–26] and Intersectionality [27–30] into their framing and analysis. CRT frames structural racism as a systemic and enduring feature of societal institutions, shaping inequities across domains such as housing, education, and healthcare [24–26]. Intersectionality emphasizes how overlapping systems of oppression, such as race, socioeconomic status, and geography, compound disadvantages for marginalized groups [27–29]. Together, they deepen the understanding of how structural disadvantage and racism intersect to produce health disparities, informing targeted public health interventions.

This can also be achieved by merging SREI scores with demographic data or modifying the index to include variables explicitly related to racial (in)equity. Longitudinal studies could enhance the understanding of causal relationships. At the same time, qualitative research could provide insights into the personal experiences of those in high SREI communities and further our understanding of the pathways leading from SREI to health adversities. Exploring the role of ethnic enclaves and community cohesion in mitigating these effects could inform more effective public health interventions.

The study underscores the importance of addressing structural determinants of health through targeted public health strategies and policies that account for racial/ethnic disparities. By incorporating social justice principles and focusing on community-specific needs, we can work toward reducing health disparities and improving outcomes for all populations. Addressing these limitations and pursuing these future directions will build upon the current findings to develop more comprehensive strategies to combat the pervasive effects of structural disadvantage and racism on maternal and child health.

## Data Availability

The data analyzed in this study are publicly available. Birth data were obtained from the National Center for Health Statistics restricted-use 2020 natality file, and access was granted under a data use agreement. The Structural Racism Effect Index (SREI) data are publicly available at https://www.sreindex.com/.
